# Early Medical Skull Surgery for Treatment of Post-Traumatic Osteomyelitis 5,000 Years Ago

**DOI:** 10.1371/journal.pone.0124790

**Published:** 2015-05-27

**Authors:** Pierpaolo Petrone, Massimo Niola, Pierpaolo Di Lorenzo, Mariano Paternoster, Vincenzo Graziano, Giuseppe Quaremba, Claudio Buccelli

**Affiliations:** 1 Laboratory of Human Osteobiology and Forensic Anthropology, Department of Advanced Biomedical Sciences, University of Naples “Federico II”, 80131, Naples, Italy; 2 Department of Advanced Biomedical Sciences, Azienda Ospedaliera Universitaria, University of Naples “Federico II”, 80131, Naples, Italy; 3 Department of Industrial Engineering, Division of Mechanics and Energetics, University of Naples “Federico II”, 80135, Naples, Italy; University of Delaware, UNITED STATES

## Abstract

Here we describe the findings of a unique example of the early techniques adopted in neurosurgery around 5000 years ago, consisting in a double well healed skull trephination associated with a post-cranial traumatic event occurring *intra vitam* to a young male from the Early Chalcolithic cemetery of Pontecagnano (South Italy, ca. 4,900 - 4,500 cal BP). Morphological, X-ray and 3D-CT scan skull-cap evaluation revealed that the main orifice was produced by scraping, obtained by clockwise rotary motion of a right-handed surgeon facing the patient, while the partial trephination was carried out by using a stone point as a drilling tool. In both cases, bone regrowth is indicative of the individual's prolonged postoperative survival and his near-complete recovery. The right femur shows a poorly healed mid-shaft fracture presumably induced by a high energy injury, and a resulting chronic osteomyelitis, affecting both femurs by hematogenous spread of the infection. Our observations on the visual and radiological features of skull and femur lesions, along with evidence on the timing of experimental bone regrowth vs. healing of lower limb fractures associated to long-term bone infections now suggest that this young man underwent a double skull trephination in order to alleviate his extremely painful condition induced by chronic osteomyelitis, which is thought to have been the cause of death.

## Introduction

Cranial trephination is the earliest form of surgical procedure widely adopted in both the Old World and the New World from the Early Neolithic [1–6]. This type of surgery, first documented in Epipaleolithic and Mesolithic hunter-gatherers [1, 7–11], most likely originated in Morocco around 11–12,000 BP [12–15]. It is still practiced at the present time among some tribes especially in Central Africa [16–18]. The first study of a trephined prehistoric skull was presented in 1867 [19]. In later decades, many other cases of skull surgery were discovered in South America [20–23], western Europe [2, 4, 7, 24–25] and in other parts of the Old World [26–32].

Trephination is the artificial removal of a bone piece of the cranial vault carried out in different ways and for different purposes [33]. Various methods were used as a surgical therapy for treatment of health problems, cranial fractures and wounds, intracranial disorders, chronic headache, brain tumors and other painful disorders [22–23, 34]. Paleopathological studies and historical sources show association of trephination with head injuries [2, 22, 35–36]. The purpose of such post-traumatic bone surgery was presumably to elevate depressed fractures, remove bone fragments and smooth broken edges, and possibly to drain epidural hematomas in order to relieve intracranial pressure [37]. The high survival rate detected suggests that ancient surgeons were able to prevent penetration of the dura mater, avoiding high risk of infection and physical damage to the underlying blood vessels, meninges and brain.

As documented in both ancient and present times, trephination has also been reported to be adopted as a magic-ritual practice [6, 16, 23, 25, 35, 37]. In these cases, defined as symbolic trephinations, only the external compact layer and possibly the spongious part were removed, without penetration of the endocranial space as in the case of complete surgical interventions [38–39]. Considering the sole quantitative aspect such as the extent of bone loss during the procedure, the terms surgical/therapeutic and symbolic could be better substituted with “complete” and “incomplete” [40].

Various congenital, developmental and acquired lesions can produce holes in the skull, and may be confused with surgical intervention [35]. A wide ranging etiology for holes in the skull can be summarized as follows: i. Congenital and developmental defects, caused by failure of ossification (e.g. enlargement of parietal foramina); ii. Pathological lesions due to disease states such as infection (e.g. syphilis or tuberculosis) or malignancy (e.g. metastatic carcinoma, bone neoplasm), but excluding trauma; iii. Surgical intervention, like cranial trepanation/trephining; iv. Traumatic lesions, usually military trauma (e.g. tangential sword cut); v. Post-mortem holes encountered in paleopathology (e.g. gnawing by animals or damage during excavation) [16, 25, 35, 39, 41].

The most frequently trepanned bone is the parietal followed by the frontal, and the left side of the skull is involved more often than the right [16]. In contrast to rare cases of children and only a small number of women involved, the vast majority of cases concern adult men. As also seen in a number of cases, the skull can be poly-trephined by two or more holes [1–2, 13, 35].

Prehistoric surgeons performed cranial perforation with various techniques such as scraping, drilling, and cutting [6, 21, 23, 34–35, 42]. Scraping, supposed to be the oldest trepanning technique, involves the use of an abrasive stone tool which is rubbed across the skull surface until a perforation is obtained. Trephinations which adopt this technique are mostly ellipsoidal and surrounded by a crater-like depression [29]. Drilling with a hard sharp stone makes conical holes of variable size, according to the sharpness of the point used, while cutting needs an incisive instrument (i.e., a flint knife) or a hard point, which allows linear, polygonal or circular craniotomies, or just a simple fusiform groove. Multiple methods can be used at the same time, resulting in more complex lesions.

Evidence of healing, indicating survival following the surgical procedure, can be classified on the basis of the degree of bone reaction to trephination: *None*—Absence of any evidence of bone regeneration, suggesting that the patient did not survive, or that the operation was performed post-mortem; *Short-term survival*—Presence of early osteoclast activity, bone necrosis, or hypervascularity, indicating survival for at least several weeks; *Long-term survival*—Evidence of extensive bone remodeling [23].

Several studies prove trephination by scraping to be the most successful in terms of survival rates [34–35]. This probably reflects the fact that adoption of stone scrapers avoided accidental penetration of the dura mater, allowing longer survival and a lower risk of infection. This has been explained by shorter duration of the operation, smaller size of lesions and the choice of surgical tool—freshly knapped flint has been shown to serve as a sterile surgical instrument—and, last but not least, the skill of the surgeon regarding the protection of the dura mater [34].

Healing processes of surgically treated ancient skulls show that in such times where anesthesia, asepsis and antibiotics were still unknown, more than 50% of trephinations were successful [25, 42–44]. If complications such as hemorrhaging, brain damage, wound infection or meningitis do not occur after craniotomy, and if primary bone healing takes place, long-term survival is often observable [1, 24–25, 32]. Referring to our specific case, it is worth mentioning an anesthetic method involving the use of natural extracts possibly adopted at that time, since finds such as "Ötzi the Iceman" seem to show that people from the Chalcolithic knew about the use of certain mushrooms as antibiotics [45].

Analysis of known cases of cranial trephination in proto-history of the Italian peninsula show 72.0% of single trephination vs. 28.0% of multiple surgical interventions (Table 1) [2, 46–47]. Long-term survival following surgical interventions is observed in most cases. Parietal bone is the preferred location of trephination.

**Table 1:** Cases of cranial trephination in the protohistory of the Italian peninsula.

Site	Period	Sex/age	Description	Technique	Survival	Ref
Abruzzi	Neolithic	♀ / Adult	Double: a. Ellipsoid / Left parietal	Scr, P-T	L-term	1
			b. Rounded / Left parietal	Scr	L-term	
Basilicata	Neolithic	♂ / Adult	Ellipsoid / Frontal, parietals	Inc	L-term	2
Latium	Neolithic	♂ / Adult	Ellipsoid / Frontal, parietals	Scr + Inc	S-term	2
Campania	Copper Age	♂ / Adult	Ovoid / Frontal, Left parietal	Scr	L-term	3
Liguria	Copper Age	♀? / Adult	Linear incisions / Parietal	Inc, P-T	S-term	2
Latium	Copper Age	♂ / Adult	Triple: a. Ellipsoid / Frontal	Burin	L-term	2
			b. Ellipsoid / Frontal	Scr	L-term	
			c. Ellipsoid / Parietal	Scr	L-term	
Tuscany	Copper Age	♀ / Adult	Sub quadrangular / Left parietal	Scr	L-term	2
Tuscany	Copper Age	♂ / Adult	Ellipsoid / Frontal, parietals	Scr	L-term	2
Sardinia	Copper Age	♂ / Adult	Ellipsoid / Frontal, parietals	Scr?	L-term	2
Sardinia	Copper Age	♂ / Adult	1. Ellipsoid / Frontal	Scr	L-term	2
		♂ / Adult	2. Ovoid / Occipital	Scr	L-term	
Tuscany	Bronze Age	♂ / Adult	1. Ellipsoid / Frontal, parietals	Scr	L-term	2
		♂ / Juvenile	2. Lanceolate / Frontal	Inc	L-term	
		♂ / Adult	3. Lanceolate / Frontal	Inc	L-term	
		? / Adult	4. Double: a. Circular /? Parietal	Scr	L-term	
			b. Circular /? Parietal	Burin	S-term	
		♂ / Child	5. Linear incision / Frontal	Inc	L-term	
Sardinia	Bronze Age	♂ / Adult	1. Circular / Lozenge	Scr	S-term	2
		♂ / Adult	2. Double: a. Ellipsoid, rounded	Scr	S-term	
			b. Ellipsoid, rounded	Scr	S-term	
		♂ / Adult	3. Double: a. Ellipsoid / Frontal	Scr	L-term	
			b. Lanceolate / Occipital	Scr	no	
		♂ / Adult	4. Quadruple: a. Ellipsoid / Frontal	Burin	L-term	
			b. Ellipsoid / Left parietal	Burin	L-term	
			c. Ellipsoid / Occipital	Burin	L-term	
			d. Circular / Left parietal	Scr	L-term	
		♀ / Adult	5. Ellipsoid / Right parietal	Scr + Inc	L-term	
		♂ / Adult	6. Triple: a. Circular / Frontal	Scr	L-term	
			b. Circular / Parietals	Scr	L-term	
			c. Circular / Parietals	Scr	S-term	
Sicily	Bronze Age	♂ / Adult	Ovoid / Right parietal	Scr	L-term	2
Lombardy	Bronze Age	♀ / Adult	Ellipsoid / Frontal	Scr	L-term	2
Friuli-V.G.	Bronze Age	♂ / Adult	Ellipsoid / Right parietal	Scr	L-term	2

Scr = scraping; Inc = incision; P-T = post-traumatic; L-term = long-term; S-term = short-term;? = undefined Ref (Reference): 1 = Capasso et al. (2002); 2 = Fornaciari et al. (1993); 3 = Present article

The present study concerns an early technological skull surgical treatment associated with severe lower limb impairment in a young adult male from the Early Chalcolithic times, around 5,000 years ago. Our observations on morphological, X-ray and 3D-CT scan skeletal features aim to shed light on the consummate skill of an early surgeon in adopting different techniques to perforate the skull successfully as a cure for a permanent disability, which was most likely the ultimate cause of death.

## Materials and Methods

All necessary permits were obtained for the described study, which complied with all relevant regulations. The Archaeological Superintendency of Salerno, Avellino, Benevento and Caserta gave permission for the custody and the study of the human skeletal materials unearthed in the 1992 excavations of the Early Chalcolithic cemetery in Pontecagnano. The human osteological materials subject of the present study are numbered PC 6589.1. The osteological materials are deposited at the Laboratory of Human Osteobiology and Forensic Anthropology, Department of Advanced Biomedical Sciences, Division of Forensic Medicine, Histology and Anatomy, II Policlinico, building 20^th^, 1^st^ floor, University of Naples “Federico II”, 5 Via Pansini—80131 Naples, Italy.

### Ethics statement

The study of specimen PC 6589.1 was approved by the Ethics Committee for Biomedical Activities of the Azienda Ospedaliera Universitaria Federico II of Naples (Protocol 560/2013, 12.18.2013).

### The Chalcolithic graves of Pontecagnano

In 1992, archaeological investigation brought to light the Early Chalcolithic cemetery of Pontecagnano (southern Italy) belonging to the Gaudo Culture, radiocarbon dated back to the 5^th^ millennium before present (ca. 4,900–4,500 cal BP) [48–49]. The graves were dug in gray volcanic tuff deposited ca. 35,000 years ago, associated with one of the huge eruptions of Mt. Vesuvius which invariably had catastrophic effects on the environment and the local inhabitants [50–52].

**Fig 1:**
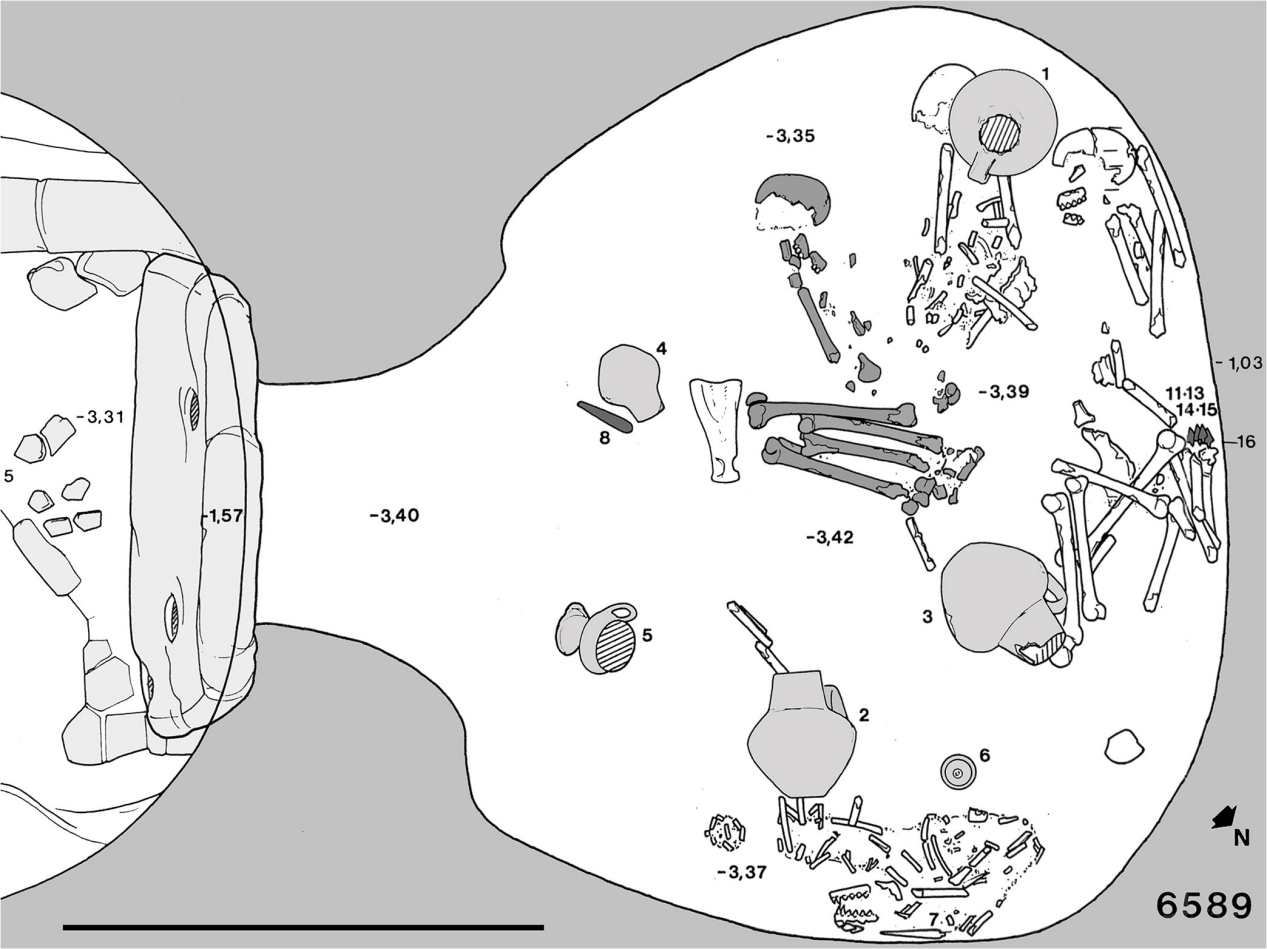
Plan of grave PC 6589. Grave 6589 consists of a single funerary chamber and a pit. The last deposition (specimen 6589.1, dark gray) is positioned at the center of the chamber, while three more skeletons are shifted (secondary burial) to the north-west side. A further group of bones belong to a dog’s skeleton. Scale bar measures 1 meter (modified from Bailo Modesti et al. 1998).

The grave structure consists of a large pit which leads to one or two cave-shaped funerary chambers. Following complex funerary customs involving the burial of the deceased, the chamber could contain one or more skeletons. According to body deposition, a distinction can be made between primary and secondary burials in relation to corpse manipulation at death or after soft tissue decomposition, respectively [53]. Secondary burials result from reduction or re-inhumation of a skeleton (or part of it). Generally, reduction involves the action of moving the bones of an individual within the same space of the primary deposition, while re-inhumation implies transfer to a different space (true secondary burials) [54]. The last buried corpse (primary deposition) is typically found lying on one side in a crouched position, while the others are reduced to small groups of bones in the same funerary chamber (reduction), or recomposed in a different one (re-inhumation) [55–56]. Tomb 6589 typically shows the last deceased (PC 6589.1) placed at the center of the chamber. The corpse is lying on its right side with flexed legs, and close to it are a large clay pot and a stone knife as grave goods, as well as a scapula of *Bos taurus* (domestic cow) (Fig 1). Analysis of the faunal remains proves that they were food offerings to accompany the deceased in the afterlife, as testified by whole-bone preservation, absence of signs of butchering, and bone groups preserving intact joint connection [53]. The bones of three more individuals are shifted to one side, together with a disarticulated skeleton of a dog. As regards taphonomic processes acting after deposition of the deceased or linked to subsequent human displacement of their bones, the skeletons are commonly represented by larger and more robust bones, most of which are partially eroded and encrusted by a thin layer of mineralized sediment as a result of periodic immersion in calcareous groundwater. In this study we present the anthropological and paleopathological analysis of the human skeletal remains from specimen PC 6589.1.

### Determination of sex and age

Sex and age at death were assessed according to standard diagnostic procedure. Following Ferembach et al. (1980) and Buikstra et al. (1994) for the attribution of sex, numerical values were assigned to each of the diagnostic skull features according to a five-point scale ranging from -2 to +2, corresponding to hyper-feminine and hyper-masculine, respectively [57–58]. Individual features were multiplied by one, two, or three, based on their significance for determination of sex. Thus, final sex assignment was the result of weighted averages based on the index of sexualization (IS), calculated according to the formula:

$$IS=\frac{\Sigma \left(\text{score x weight}\right)}{\Sigma \text{weight}}$$

Positive IS values identify a specimen as male, while negative IS values identify a specimen as female. If the IS score is zero or approaching zero, the sex of the specimen must be regarded as uncertain. Recommendations of Rösing et al. (2007) were also considered [59]. Poor preservation of pelvis bones did not allow any reliable sexing determination. Individual’s age at death was roughly determined using the wearing stage of teeth according to Miles (1963) and Lovejoy (1985) [60–61]. The stage of ectocranial suture closure based on the method of Meindl and Lovejoy (1985) was also evaluated [62].

### Paleopathological analysis

Specimen 6589.1 was examined for bone anomalies in size, shape and topography [33, 39]. In addition to gross anatomy, we conducted a detailed radiological investigation adopting digital radiography (Villa Mercury 332, Kodak Direct View CR 850; Naples, Italy) and 3D computed tomography (SCANORA CT-3D, Soredex, digital X-ray system; Naples, Italy) [63]. For differential diagnosis of the skull lesions, the guidelines of Aufderheide et al. (1997) and Steinbock (1976) were followed [39, 41]. The suggestions of Wakely (1993) were also taken into account [64]. Distinctive features of both skull lesions as regards difference in shape, size, location and postsurgical bone reaction were evaluated and compared with several conditions which simulate trephination, as summarized in Table 2 and later discussed. The possible involvement of taphonomic agents in causing the skull lesions was discounted using the methods and terminology of Lyman (2001) [65] (Table 3). Considering number, location and morphology of the skull lesions, the described bone assemblage attributes lead us to rule out taphonomic agents such as gnawing, weathering, or further chemical and/or physical phenomena in causing the lesions. Diagenetic processes mainly arising from the wet alkaline burial environment and subaerial/surface exposure (corpse buried in an empty space) in a long-lasting preserved context (vault-shaped grave, deep within the soil) may better explain the scattered superficial bone decay and covering by sediment, partial crushing and incompleteness of skeleton 6589.1. According to the Arbeitsgemeinschaft für Osteosynthesefragen classification (AO), for the diagnosis of the femoral shaft fracture the guidelines of Salminen (2005) and Müller et al. (1990) were utilized [66–67]. The femoral diaphyseal abnormalities were assessed following the standards and criteria of Ortner (2003) and Aufderheide et al. (1997) [33, 39]. The UTMB (University of Texas Medical Branch) classification of adult osteomyelitis reported in Cierny et al. (1985) was also adopted [68].

**Table 2:** Differential diagnosis for skull trephination (modified from Steinbock, 1976, Aufderheide et al., 1997, and Ortner, 2003).

Skull lesions	Lesion features	Features of 6589.1 major skull lesion	Features of 6589.1 minor skull lesion
1. *Enlarged parietal foramina* (Biparietal congenital perforation)	Oval, symmetrical lesions; irregular shape and borders.	Ellipsoidal, volcano-like shape; lesion not symmetrical.	Round, conical shape; lesion not symmetrical.
2. *Cranial dysraphism* (Congenital herniation)	Sharply defined borders; asymmetric shape.	Beveled borders; symmetric shape.	Beveled borders; symmetric shape.
3. *Tangential sword cuts*	Inner table defects size exceeds outer; sharply defined borders.	Outer table defect size exceeds inner.	Defect involving the sole outer table.
4. *Comminuted fractures*	Much more irregular; possible retaining of fracture line.	Regular; absence of fracture lines.	Regular; absence of fracture lines.
5. *Metastatic carcinoma; myeloma*	Irregular, often multiple, destructive; no reactive borders, erosion, osteolysis.	Regular, well defined, new bone formation; lone in its cranial district.	Regular, defined, new bone formation; lone in its cranial district.
6. *Bone neoplasm*	Destructive. Differently from trauma, trephination and cauterization, benign tumors do not produce synostosis.	New bone formation; advanced synostosis.	New bone formation; advanced synostosis.
7. *Infections* (Syphilis, tuberculosis, mycoses)	Irregular, multiple, osteolysis; extensive sclerotic healing.	Regular, well defined; new bone formation; lone in its cranial district.	Regular, defined; new bone formation; lone in its cranial district.
8. *Nonspecific infections*	New bone response, irregular, diffuse.	Regular, well defined.	Regular, defined.
9. *Parietal bone osteopenia* (Biparietal thinning)	Depression not sharply demarcated, symmetrical; thinning of the outer table.	Regular, well defined, not symmetrical; thick outer table.	Regular, defined, not symmetrical; thick outer table.
10. *Postmortem alterations* (Natural taphonomic agents)	Erosion by stone abrasion, acid soils, animal effects; irregular.	Regular, new bone formation; close environment (burial structure).	Regular, new bone formation; close environment (burial structure).
11. *Excavation injuries* (Anthropic actions)	Accidental holes by sharp instruments or picks; irregular, sharp edges.	Regular, beveled borders; absence of fractures.	Regular, beveled borders; absence of fractures, intact inner table.

**Table 3:** Taphonomic (biological, geological, human) agents possibly involved in causing the skull lesions.

Taphonomic agents	Bone assemblage attributes
a. position	deep (few meters) from surface
b. depositional unit attributes	laying on tuffaceous fine textured sediment
c. anatomical distribution of damage	proximal and/or distal
d. weathering	low, flaking of outer surface
e. gnawing damage	absent
f. polish/abrasion/corrosion	random superficial fine erosion
g. distortion/deformation	absent
h. fractures, crushing	random, mostly affecting smaller, more fragile bones
i. completeness	incomplete skeleton, larger bones articulated with other bones *in situ*

## Results

Sex of the specimen 6589.1 was determined by score and weighting evaluation of the following cranial traits: glabella (marked, +1), mastoid process (very large, +2), nuchal plane (marked, +1), zygomatic arch (very thick and high, +2), superciliary arch (very marked, +1), frontal and parietal eminences (missing, +2), external occipital protuberance (medium, 0), shape of the forehead (strongly inclined, +2), zygomatic bone (very high, +2), supraorbital margin (very rounded, +2), chin (triangular, +2) and mandibular angle (very prominent, +2). The resulting weighted average of 1.65 (IS) led the specimen to be assigned as male [57–58].

**Fig 2:**
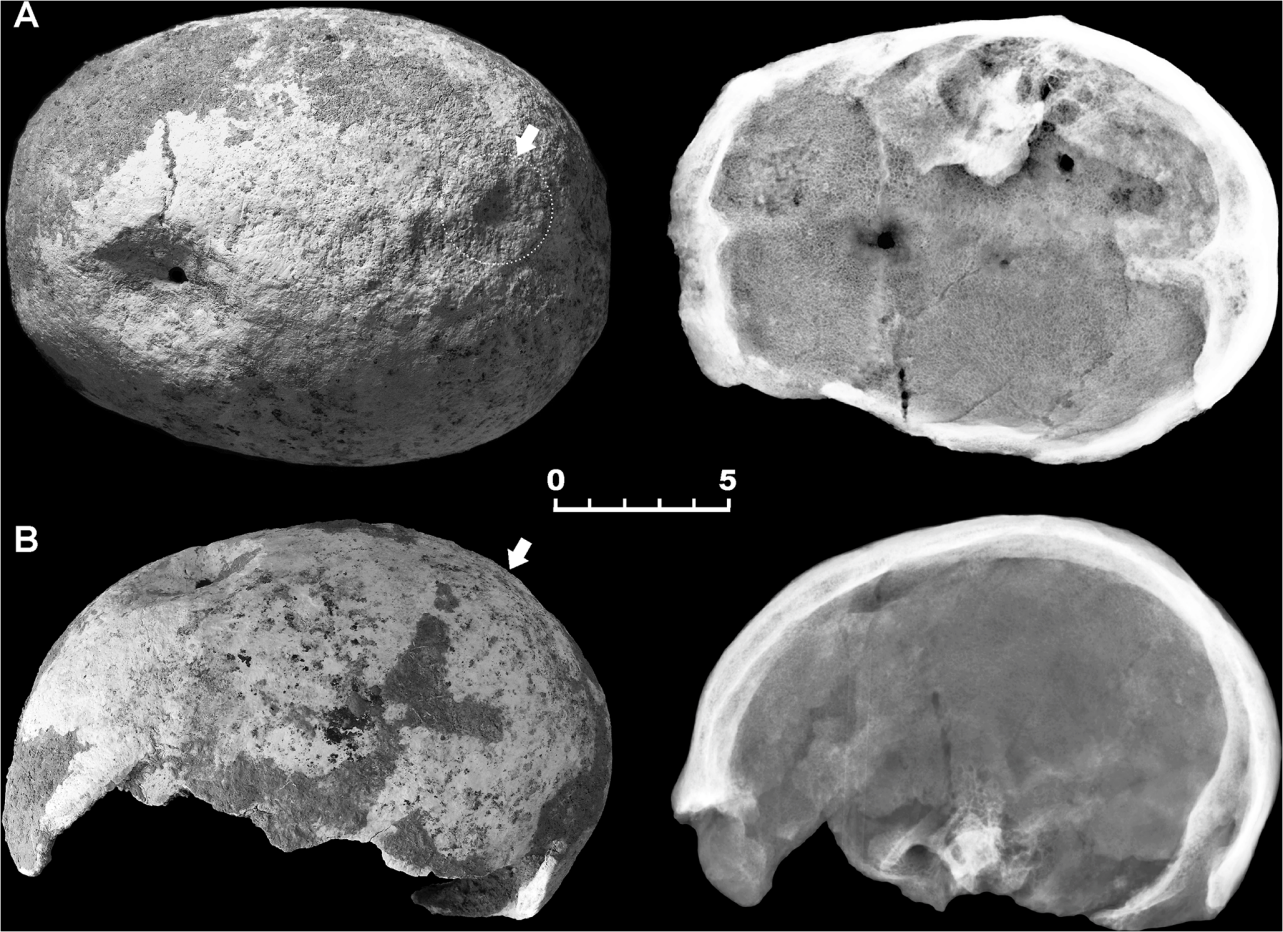
Double skull trephination affecting specimen 6589.1. Superior (A) and lateral (B) view and corresponding X-ray images of the skull-cap. Wide crater-like elliptical depression across the bregma, showing well-defined, irregular borders (A, B). Gross morphology and X-ray images show healing, new bone being more radiolucent and with less mature architecture compared with surrounding old bone. A further minor depression in located close to the lambda (arrows) (A, B). Scale bar equals 5 cm.

**Fig 3:**
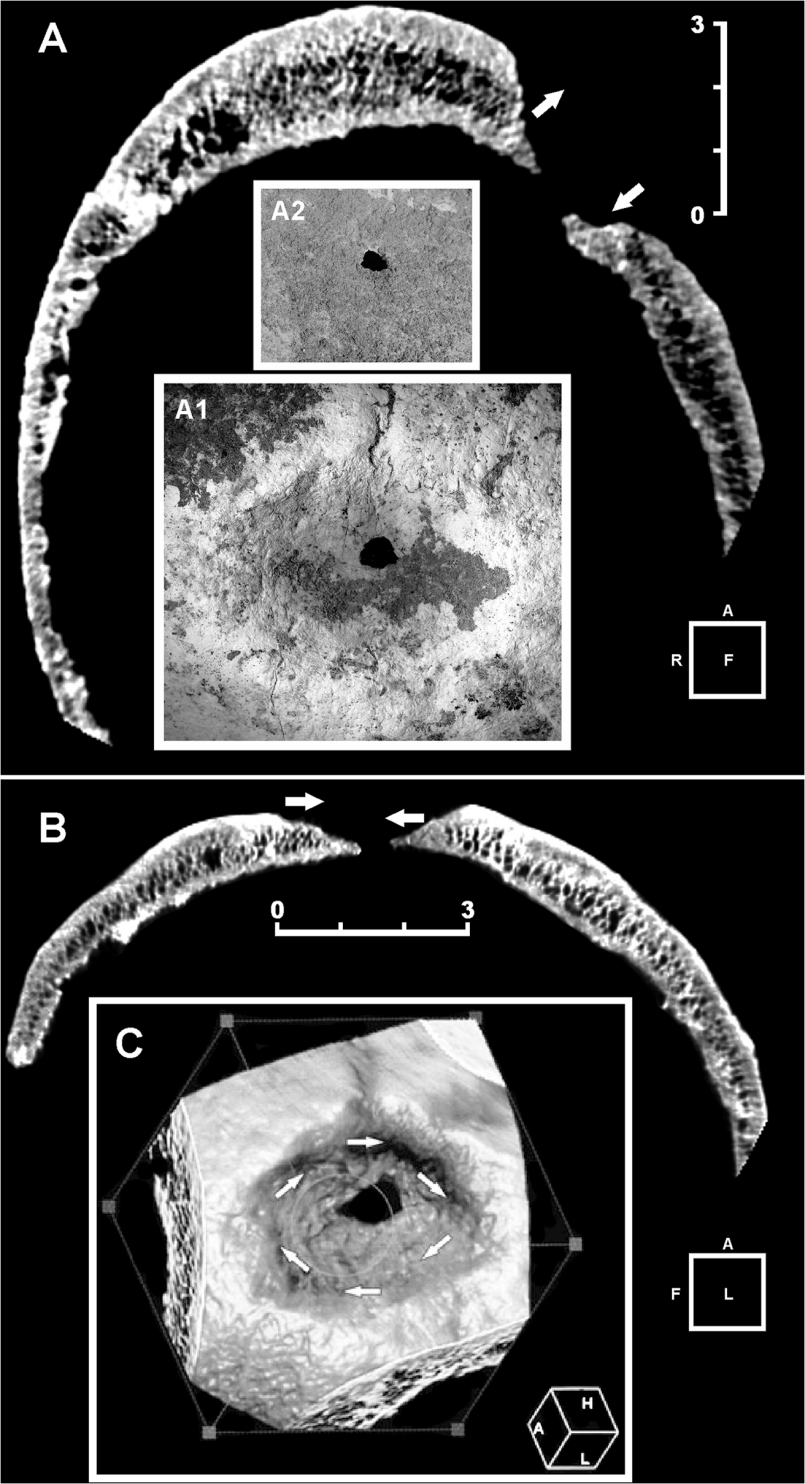
Three-dimensional (3D) computed tomography (CT) scan of the main skull trephination. Frontal (A) and sagittal (B) plane CT scan. Extensive and irregular bone reconstruction is apparent. No evidence is seen of incomplete healing processes as in the case of complication by infection. Note the different angle at the top/bottom (frontal plane) (A) and front/back (sagittal plane) (B) bone around the hole, indicative of right-handed clockwise rotation applied during the action of scraping (arrows). Detail of the main lesion, whose edges show active regenerative bone processes (A1). Endocranial aspect of the lesion. The bone surrounding the hole is intact (A2). 3D reconstruction of the outer aspect of the trepanned cranial vault. The arrows show the clockwise rotation movement (C). Scale bars measure 3 cm.

The skeleton of the 25-year-old male 6589.1 shows multiple lesions. An ellipsoidal depression involving both the left parietal and the frontal bones perforates the bone near bregma at the fronto-parietal junction (Fig 2A and 2B). This crater-like lesion measures a maximum of 49.0 mm anteroposteriorly by 39.5 mm mediolaterally at the ectocranial surface (Fig 3A1). The depression is characterized by raised and remodeled edges, an oblique orientation of the walls testified by a thickness gradually decreasing from the borders towards the central opening, and a central irregular foramen with beveled rather than sharp margins measuring ca. 5.9 X 5.1 mm (Fig 3A and 3B). The inner bone surface around the hole is intact (Fig 3A2).

**Fig 4:**
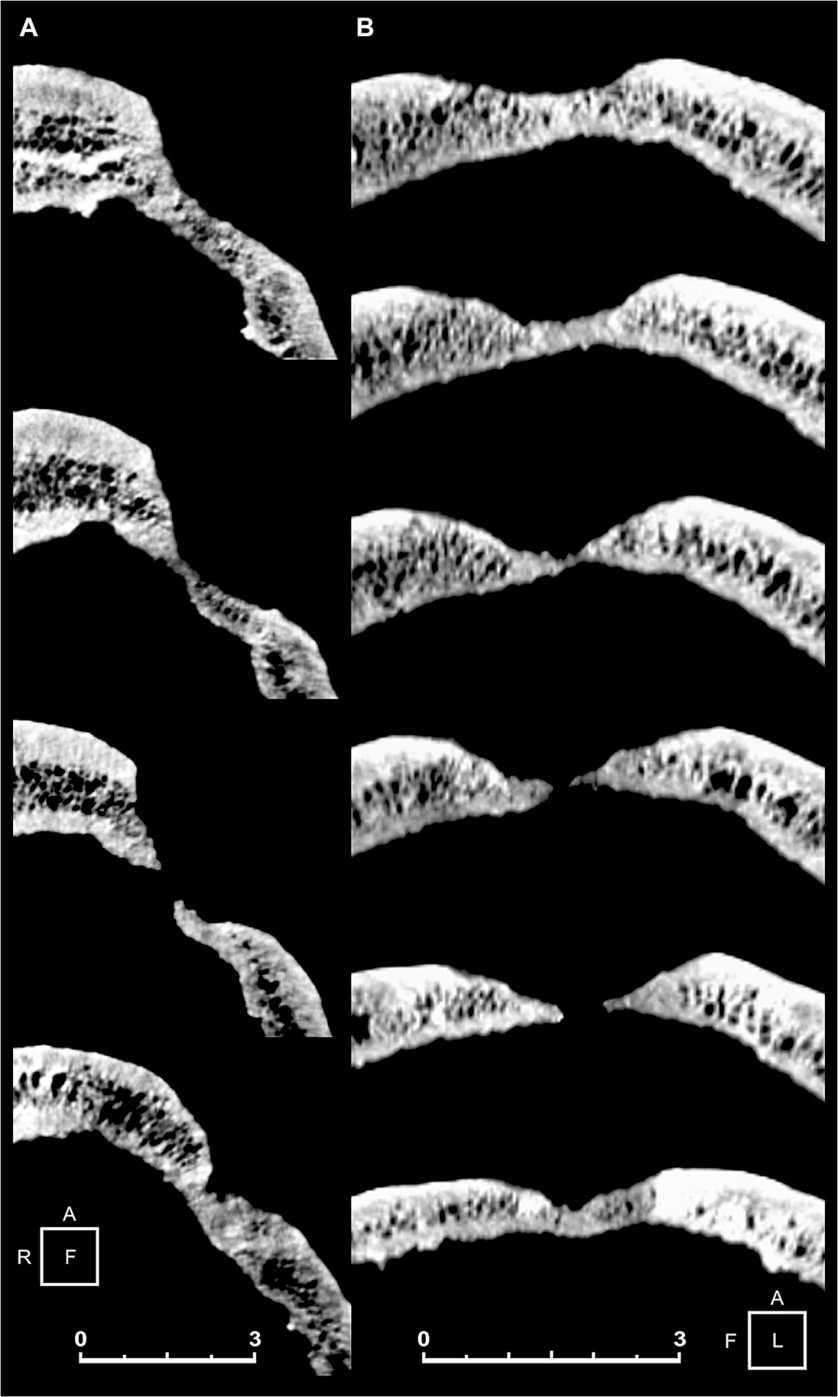
Three-dimensional (3D) computed tomography (CT) scan slices of the main skull trephination. Coronal (A) and sagittal (B) plane CT scan cross section sequence. Note the oblique orientation of the hole walls, and the defect edges remodeled into one compact bone layer, as a result of the loss of diploic structures. The smoothed, beveled edges are also indicative of bone regrowth. Scale bars measure 3 cm.

On visual, X-ray and 3D-CT scan examination marked features are the oblique orientation of the hole walls, the defect edges remodeled into one compact bone layer, and the resulting loss of visible diploic structures. As a general feature, the disturbed bone appears to be more compact than the rest of the skull (Fig 2A and 2B and 3A and 3B). The smoothed, albeit slightly uneven, edges with beveling indicate the regrowth of bone, as apparent from examination of both the peripheral part of the depression and that close to the center, characterized by reactive new bone formation and substantial bone remodeling [29, 69]. Considerable osseous regeneration is also testified by fusion of the outer and inner bone layers at the defect margins and disappearing of the diploic structure, as evident from 3D-CT scan slice sequence both in the coronal and the sagittal projections (Fig 4A and 4B). This would indicate the individual's prolonged postoperative survival, since skull bone regenerates slower than long bones [69–70].

**Fig 5:**
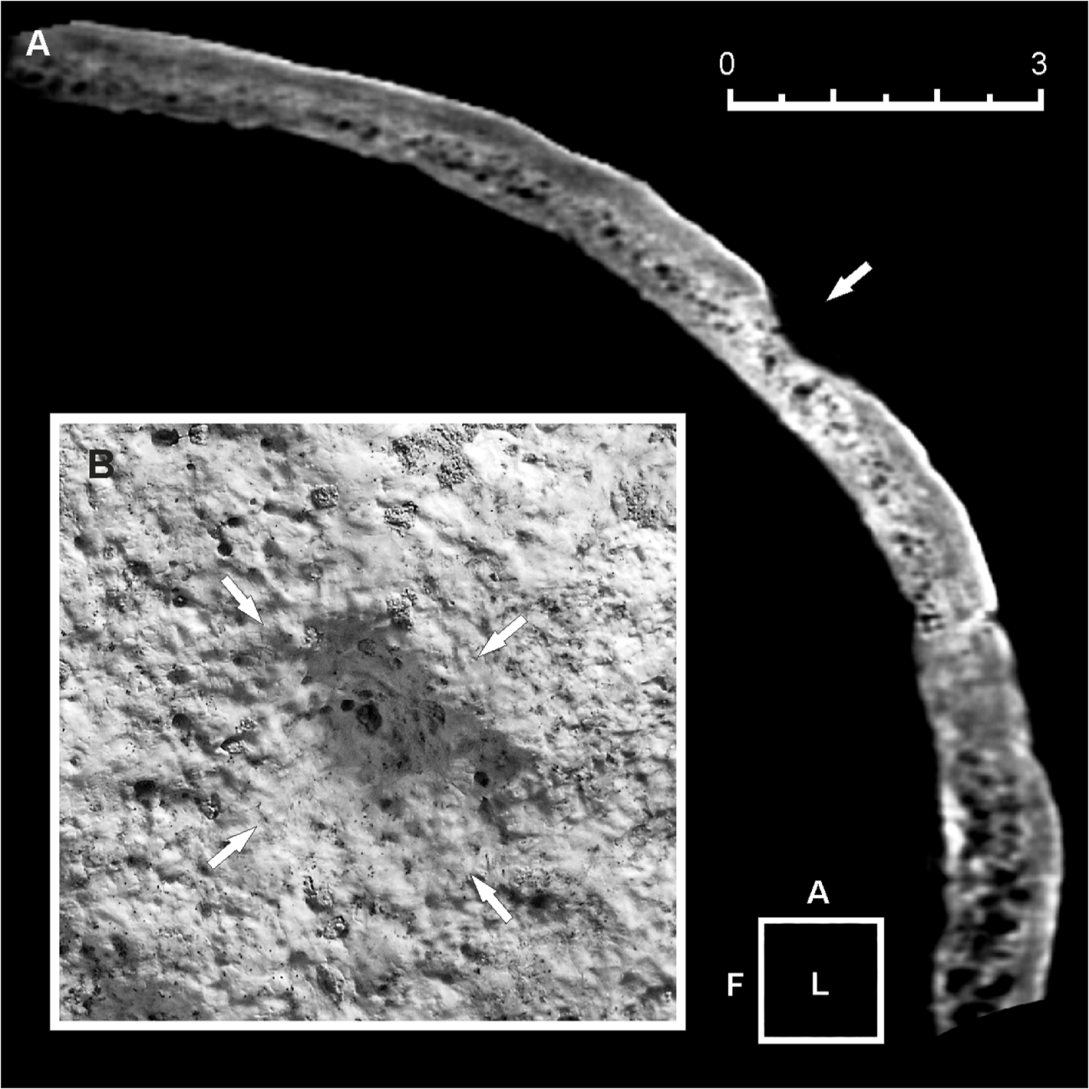
Three-dimensional (3D) computed tomography (CT) scan of the minor skull trephination. (A, B) Sagittal plane CT scan (A). Only the external cortical layer of the bone is affected (arrow). Detail of the cone-shaped lesion, identified as an incomplete trephination with a possible ritual purpose (B). Bone remodeling, hypervascularity and pitting of the ectocranial surface suggest an advanced phase of the bone healing process. Scale bar measures 3 cm.

A second smaller conical round depression is placed close to the sagittal suture in proximity to lambda, where the right parietal foramen is commonly located (Fig 2A). The hole measures ca. 4.9 X 5.8 mm in size and ca. 15.3 mm in depth. Unlike the larger lesion, only the cortical layer of the vault is affected, while the endocranial compact bone has not been reached (Fig 5A). The outward appearance of the orifice, characterized by extensive bone remodeling as testified by hypervascularity and diffuse pitting of the bone surface, also suggests in this case an advanced phase of the regenerative process (Fig 5B).

**Fig 6:**
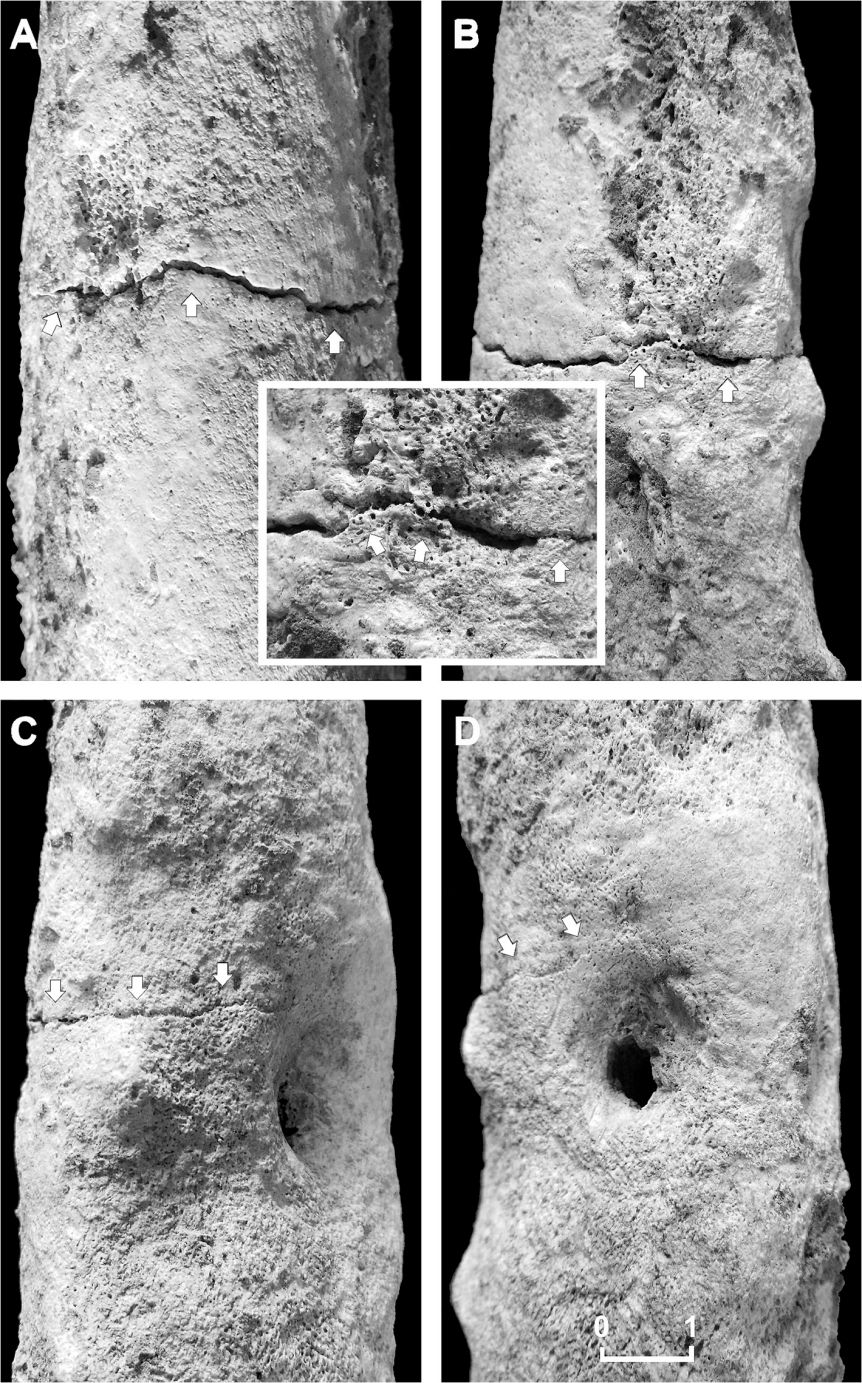
Details of the femoral nonunion fracture. Anterior (A), medial (B), posterior (C) and lateral (D) views of the middle shaft of the right femur showing a transverse simple linear fracture (arrows), characterized by unhealed margins and lack of bone callus regeneration (A-D). Wide cloaca opening, surrounded by reactive bone (D). Detail of few bone bridges (box) on the medial aspect of the lesion, indicative of poor bone repair. Scale bar equals 1 cm.

**Fig 7:**
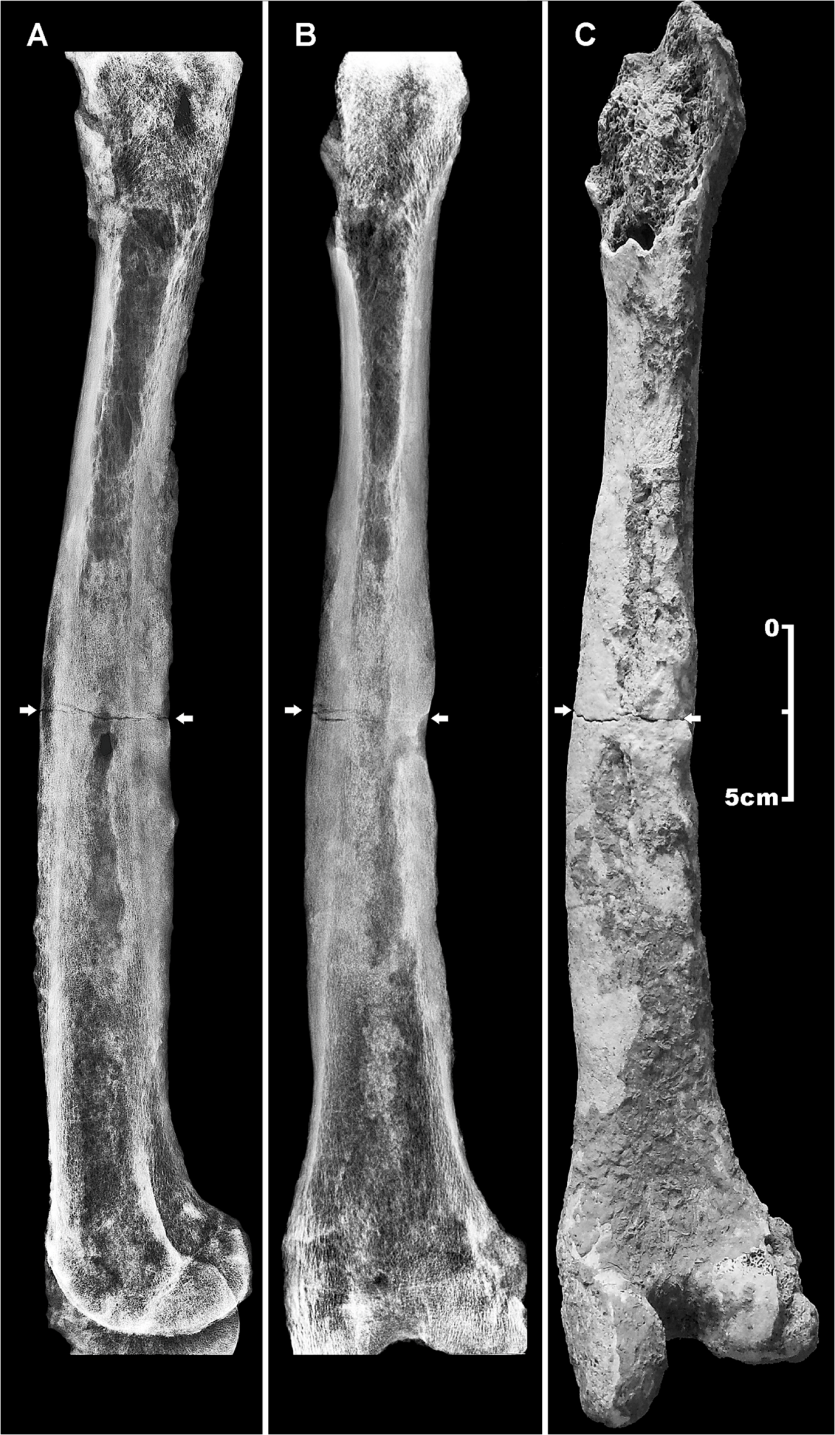
Traumatic and osteomyelitic femoral lesions. Latero-lateral (A) and antero-posterior (B) X-ray images, and posterior view (C) of the right femur (A, B). Diffuse periosteal new bone apposition (*involucrum*) testified by a more radiolucent enlarged cortical bone layer, a reduced medullary cavity, and a cloaca drainage canal, diagnostic of long-term chronic osteomyelitis (C). Abnormal size of the femoral shaft induced by periosteal new bone apposition. At the mid-shaft, the fracture line and the cloaca opening are evident (arrows). Scale bar equals 5 cm.

**Fig 8:**
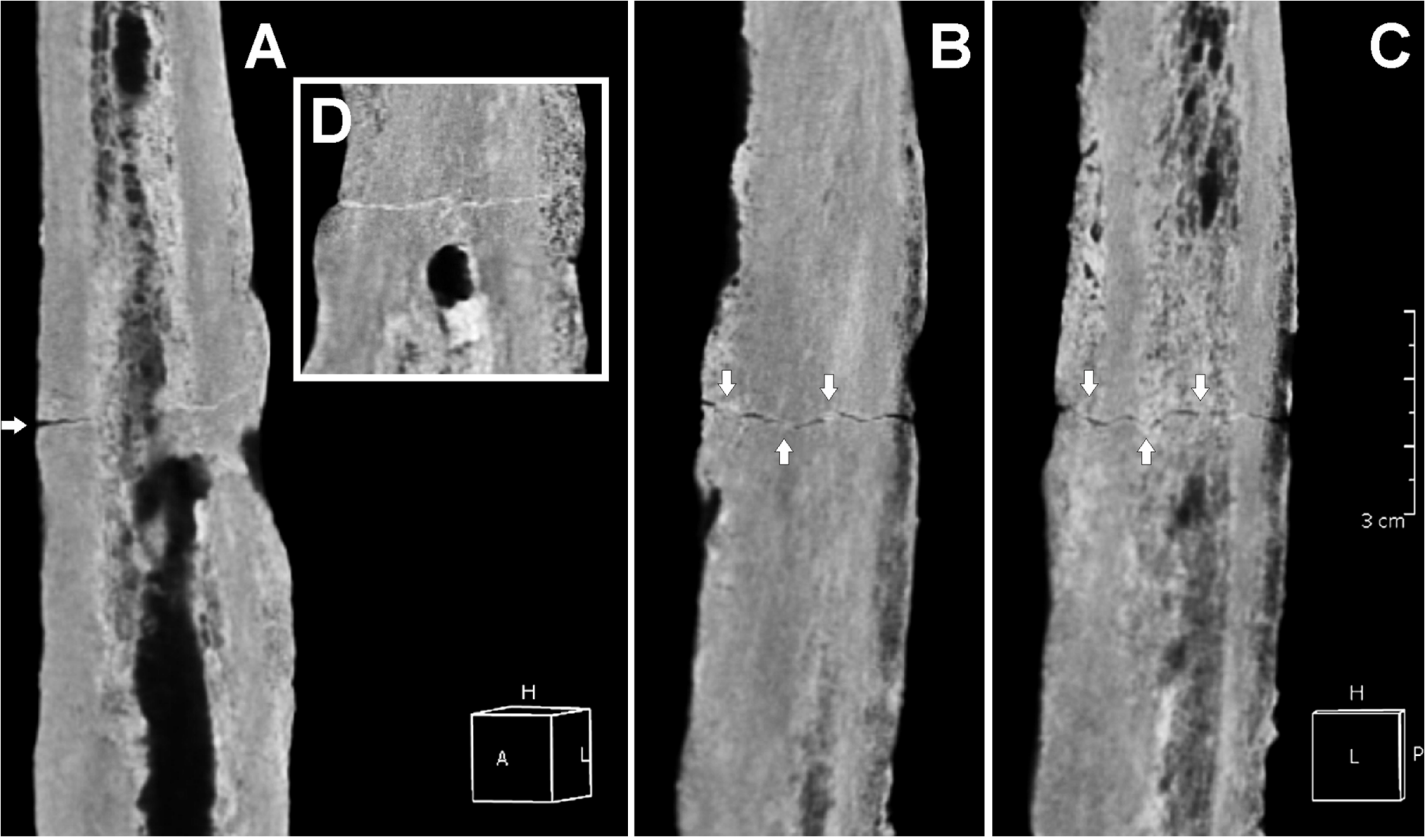
Three-dimensional (3D) computed tomography (CT) scan of the femoral lesion. Posterior (A), anterior (B), and medial (C) views of the transverse simple linear fracture. New bone apposition (*involucrum*) is evident. The bone lesion is characterized by poor healing (arrows) (D). Detail of the cloaca opening (box) and, above, the uninterrupted fracture line. Scale bar equals 3 cm.

Further examination of post-cranial skeletal elements show the right femur to be affected by a transverse incomplete, non-comminuted linear fracture, a horizontal line going across the middle shaft (Fig 6A–6D). Referring to the AO classification, the femur lesion was classified as a simple, Type A, transverse (angle of less than 30 degrees, measured by the angle between a line perpendicular to the long axis of the femur and the main fracture line), grade III fracture (fragmentation over 50% of the width of the femoral shaft) [67]. Such a lesion may occur as a consequence of a direct fracturing force, depending on its magnitude, direction, and nature of load [66]. In the present case we may suppose that the individual suffered an open fracture, possibly from an outside force, with interruption of skin continuity at the site of the lesion, which resulted in bone marrow infection. A fatigue fracture induced from prolonged, excessive or repetitive physical activity seems less likely, femoral shaft lesions being commonly non-comminuted of oblique or oblique-transverse type. Plain radiology (Fig 7A–7C) and CT scan (Fig 8A–8D) revealed the lack of both substantial fracture healing and bone callus formation, thus resembling a nonunion fracture.

In addition, the femoral shaft shows new periosteal bone apposition (*involucrum*) and enlarged bone cortical layer, loss of cancellous bone architecture, and a drainage canal (cloaca) close to the fracture, features diagnostic of osteomyelitis [33, 39] (Fig 7A and 7B and 8A–8C). On the basis of the current clinical staging system for adult osteomyelitis, the lesion was classified as anatomic Type IV diffuse osteomyelitis [68]. The considerable diaphyseal enlargement and the formation of a cloaca opening characterized by smooth compact walls and penetration into the narrow space are symptomatic of a chronic inflammatory process [39]. The left femur is also involved, albeit to a lesser extent.

## Discussion

Several studies have described the importance of applying 3D computed tomography imaging in detecting bone paleopathology [63]. Analysis of skeletal features of specimen 6589.1 shows convincing evidence of *intra vitam* complex skull trephination, thus providing new insights into prehistoric neurosurgical technology. Detailed X-ray and 3D-CT scan skull-cap evaluation of the main lesion revealed that in this case the orifice was most probably produced by scraping with a sharp stone tool rather than by boring or sawing to perforate the skull [34, 71]. The oval, crater-like shape, the gradual bone thinning toward the center and its location strongly suggest that the orifice was obtained by rotary motion of a right-handed surgeon facing the patient (Fig 3A and 3B). The different angle resulting from the variation in thickness gradient between external and internal surfaces of the cranial vault at the top/bottom (frontal plane) and front/back (sagittal plane) bone surrounding the hole was most likely produced by clockwise rotation applied during scraping (Fig 3C). In contrast, the incomplete second trephination seems carried out by drilling with a stone point as a tool, which produced a round, cone-shaped hole, involving only the external cortical layer (Fig 5A and 5B).

The evidence of bone regeneration provides the strongest argument for trephination, since very few other perforations of the skull involve regrowth of damaged bone. In both trephinations the regrowth is documented by the dense diploe and the beveled but uneven edges, resulting in a raised volcano-like area of bone surrounding the lesion. The remodeled defect margin of the opening characterized by one compact bone layer and the concomitant loss of diploic structures (Fig 7A and 7B and 8A–8C), as well as an elongated opening with oblique orientation of the hole walls are distinctive of trephination [6, 16, 27]. Such sloping inclination reveals the need to control the surgical process and avoid damage to the dura mater [26]. The scattered pitting, particularly evident around the minor perforation (Fig 5B), suggests osteitis, a common finding in trephined skulls [33]. As supported by radiological and visual rendering, the apparent absence of signs of recent bone healing and the substantial remodeling of the lesions confirm the individual’s survival after both surgical operations and his near-complete recovery, even in the case of the major trephination defect, whose incomplete obliteration testifies a long-standing regenerative process as commonly observed in modern post-surgical skull healing [69]. Examples of trephinations with no associated signs of trauma are well documented from the Neolithic onward [4].

As regards possible differential diagnosis (Table 2), in our case conditions such as congenital and developmental defects, and pathological or traumatic lesions of the skull are not verifiable (33, 39, 41). In particular, we may rule out enlarged parietal foramina due to the symmetrical nature of this congenital lesion and the irregular shape and borders. Cranial dysraphism can also be excluded due to the asymmetric shape of the hole, its sharply defined borders, and the marked porosity limited to the bone area bordering the lesion. Furthermore, because of the hereditary nature of both the previous anomalies, it seems unlikely that only one individual in the Chalcolithic population of Pontecagnano would display this condition (19 preserved skulls were examined from 52 specimens). The typical osteoporotic manifestations seen in byparietal thinning as porous and thinned area around the defect appear in contrast with dense thick bone surrounding the skull lesions. Pathological conditions such as metastatic carcinoma, myeloma, bone neoplasm, and infections can cause lytic, irregular, often multiple bone defects in the skull apparently similar to unhealed trephined openings. However, the evidence of reactive osseous regrowth around the lesions, typical of healing after successful surgical trephination, clearly rules out non-traumatic anomalies as congenital, developmental or pathological conditions, commonly characterized by failure of ossification [33, 35, 39]. Considering trauma (i.e. blunt trauma, comminuted fractures) as the possible cause of one or both skull lesions, the concentric groove morphology and the absence of radiating or concentric fractures provide evidence that the holes are not due to an injury, as partly suggested by the lack of evidence of osteomyelitis affecting the skull-cap [33]. As previously described, apart from some scattered superficial bone erosion induced by burial wet environment, considering number, location and morphology of the lesions, other postmortem alterations induced by natural taphonomic agents or accidental holes made with sharp instruments or picks during excavation can also be excluded.

Despite the several perforative lesions of the skull that may mimic trephination defects, several observations on trephination conditions derived from present-day cases [69] can be made: i. Since new bone formation seems to occur only minimally on the skull, trephination defects hardly ever show complete obliteration; ii. Remodeling by osteoclastic resorption of the defect margins at the trephination site leads to a smoothing of the initially sharp edges. A distinctive feature is the loss of the typical layering of the skull bone at the defect margins, in particular the loss of the diploic structure. Finally, the remodeled defect margin consists of only one compact bone layer, and the internal and external tabula can no longer be distinguished; iii. Healing originates in the outer table; iv. These remodeling processes follow a definite time course: defects with rounded, smoothed margins and a loss of the diploic skull bone structure must have survived for at least several months, or years. Finally, comparative evaluation of the previous conditions with the features detected from both skull lesions of specimen 6589.1 regarding location, shape, size, and postsurgical reaction of the ectocranial surface point to surgical treatment by trephination.

Since neither bone nor brain possess pain receptors, trephination should not have been particularly traumatic or painful after the scalp and soft tissue had been pierced, but the considerable hemorrhage occurring during the surgery from exposed spongy bone had in some way to be controlled. As long ago as 3,000 BCE, Egyptian papyri describe the practice of fresh meat application to wounds to decrease bleeding due to its hemostatic properties [72]. As reported on clay tablets from ancient Mesopotamia, a specific surgical procedure mentions the knife scraping the skull of the patient, and the use of plants to stop bleeding. Ancient Greek surgeons applied cautery in hemostasis, bending and casting [73]. Nowadays, surgeons use bone wax, a nonabsorbable mixture of beeswax, paraffin, isopropyl palmitate, and a wax-softening agent, to stop bleeding [74]. In our case, due to the location of trephining, we may consider digital pressure to be adopted as the most simple hemostatic procedure [27], with bleeding controlled by applying plant extracts or mixtures of crushed leaves. This procedure, practiced by surgeons in prehispanic South America [75], is still adopted in modern Kenya [37].

Even if no evidence of trauma or infection is detectable by X-ray and CT-scan skull and teeth evaluation, as in most cases in which communication between the cranial cavity and the environment is created we hypothesize a therapeutic purpose for the surgical treatment of the main trephination [24]. As to the minor depression, given the incomplete nature of the surgical intervention, its morphology and location, a symbolic purpose could be suggested [25, 38, 40]. Symbolic or incomplete trephinations were performed mainly on adults, mostly males, often along sagittal and coronal sutures, the trepanned shape being round or oval [38, 40].

Due to the fact that paleopathologists are limited to skeletal evidence alone, the specific motivation for most trephinations is unknown. In our case, we assume that the reason for the skull surgery performed on this young adult male was a traumatic event occurring during his life, consisting in a simple, non-comminuted, purely transverse fracture [67], complicated by a post-traumatic infection. As reported by a recent clinical epidemiological study of the femoral shaft fractures, this is the most common traumatic fracture type of femoral shaft [66]. The line of breakage frequently resulting from direct violence is usually transverse, and caused by the most common injury mechanism, bending load. A bending load applied to a diaphyseal bone results in transverse fractures where the location of a soft-tissue hinge is on the concave side; in the femur, the femoral shaft fails first under tensile strain that is maximal on the anterolateral aspect of the femoral shaft. This condition is likely to match our case, the mid-shaft transverse fracture being more apparent on both the anterior and medial femoral aspects (Fig 6A–6D). Instead, fractures from indirect impact are usually oblique, and caused by axial compression with bending and torsion. Fractures due to muscular action are characterized as spiral, and caused by torsion load. Oblique and spiral fractures are frequently compound fractures.

Abnormal bone remodeling of the right femoral shaft, including deformity with enlargement of the entire infected bone due to reactive new bone formation (*involucrum*), a more radiolucent thickened diaphyseal cortical layer, reduced medullary cavity, and the formation of a drainage canal (cloaca opening) are diagnostic of chronic osteomyelitis (Fig 7A–7C and 8A–8C) [33, 39, 68]. Chronic cases, which can evolve over months or years, are characterized by the persistence of microorganisms and the extensive spread of the infection through the bone, inciting osteoblast activity, expanding the contours of the bone and reducing the size of the medullary cavity [76]. In our case, the long-standing onset of the pathological process is confirmed by the osteomyelitic minor involvement of the left femur, due to a secondary hematogenous spread of the infection.

Osteomyelitis is a difficult-to-treat infection characterized by progressive inflammatory destruction of the infected bone and new apposition of bone at the site of infection [77]. The radiological findings are bone destruction, reactive new bone formation, bone necrosis, and the typical lytic areas in combination with other areas of increased bone density [39]. In adults osteomyelitis is usually a complication of open wounds involving the bone, either from fractures, surgery or both. This infection may develop as well in a non-injured bone after bacteremia. Thus the bacteria can reach the skeleton by an exogenous route, or by the hematogenous route from a remote septic focus [33, 39]. Osteomyelitis is almost always caused by pus-producing microorganisms like pyogenic bacteria and mycobacteria, and is thus called suppurative or pyogenic osteomyelitis [70]. *Staphylococcus aureus* is the microorganism which most frequently causes both post-traumatic and hematogenous osteomyelitis [78]. The disease, and particularly its hematogenous form, greatly predilects the long bones of the extremities, involving as well the medullary cavity. Hematogenous infection by a low virulence organism can produce chronic osteomyelitis from the onset of infection [39]. Osteomyelitis is one of the most severe complications that can arise following bone trauma, and even in recent times the patient can never be sure that it will heal completely [79]. In spite of appropriate combined medical and surgical therapies up to 30% of osteomyelitis infections become chronic, causing morbidity and mortality [80]. Hematogenous acute osteomyelitis can cause septicemia and kill the patient even before chronic osteomyelitis can develop, representing a potentially major cause of death in the pre-antibiotic era [76].

As is also evident in our case, a nonunion may arise if a bone fracture does not heal correctly. If infection occurs, the result may be an infected acute fracture producing deformity and/or complete failure for the bone ends to mend [81]. The most common reason for infection such as osteomyelitis following bone trauma is an open fracture. If a bone breaks in such a way that bone fragments stick out through the skin or a wound penetrates through soft tissue coverings down to the broken bone, the medullar cavity can be exposed to bacterial contamination. Open fractures have a higher risk of complications—especially infections—and take longer to heal.

Although infection and the severity of bone trauma are important deterrents to normal fracture healing, instability is the most common cause of a nonunion [82]. A nonunion occurs when a bone does not heal within six to nine months after a break or fracture [83]. Several factors were found to have an adverse effect on nonunion (bone fractures) healing, including advanced patient age, presence of osteomyelitis, duration of nonunion and inadequate stabilization after the break/fracture. Existing guidelines recommend emergency surgical debridement of open fractures within a few hours after injury [84]. In the present case, the delayed process of healing of the femoral shaft fracture and the subsequent nonunion of bone most likely induced by the osteomyelitic infective process following the trauma is testified by complete absence of a primary bone callus, which normally takes six weeks to develop [70].

Our study suggests that this young man initially suffered a mid-diaphyseal fracture, possibly caused by a mechanical injury, which induced post-traumatic chronic osteomyelitis consistent with an infected femoral nonunion. Femoral shaft fractures, commonly occurring among younger adult males, typically exposed to high energy injuries, are usually severe and often associated with serious blood loss [85]. Epidemiological clinical data prove that 50% of nonunion of femoral shaft fractures do not heal in treated patients, while the mean duration of follow-up after establishment of nonunion was 62 months, with 24.5 months being the mean duration from fracture to care [83]. In contrast, estimates of bone remodeling in normal human subjects show a total mineralization phase of 17 to 20 weeks [86], while skull bone regeneration in primates was completed after 21 weeks [87]. A further indirect indication on the timing of occurrence of both lesions detected on the skull and lower extremities of specimen 6589.1 is possibly given by epidemiologic quantitative assessment of the effect of head injury on fracture healing [88–89]. In patients with traumatic head injuries and fractures of long bones it is often clinically observed that the rate of bone healing and extent of callus formation are increased, leading to rapid union [90]. Experimental biomechanical tests and finite element analysis also suggest that head trauma contributes to fracture healing [91].

The different degree and timing of skull vs. lower limb bone repair allow us to hypothesize that individual 6589.1 underwent a complex surgical intervention by double skull trephination as a consequence of a permanently invalidating trauma consistent with chronic osteomyelitis induced by femur fracture and infected bone nonunion. Nonunion fractures complicated by osteomyelitis represent a group of more severe injuries which predispose patients to a poor outcome [83].

## Conclusions

Here we described two bone lesions that we detected as surgical trephinations of the skullcap in a Chalcolithic young adult male. Detailed analysis was performed to discern some aspects of the operator’s technique and examine the individual’s post-operative survival, as supported by evidence of perilesional bone healing. A further lesion was documented in the lower limb, consisting in a chronic post-traumatic osteomyelitis secondary to hematogenous spread of a bone marrow infection, most likely induced by the poorly healed mid-shaft fracture of the right femur. The near-complete bone healing after skull trephination resulting in long-term survival, and the concomitant persistent lower limb infection strongly suggest that such a chronic disabling disease was the reason for the double surgery, and most probably the ultimate cause of death of the individual. We may reasonably assume that the purpose of trephination was to free him from his extremely painful condition resulting in permanent disability, possibly identified with an evil spirit. In this respect, this case is a unique example of prehistoric neurosurgery, which documents in detail the consummate skill of an early surgeon in successfully adopting different techniques to perforate the skull as treatment for an invalidating body trauma.
